# Supplementary material for: Are carbon emissions trading and green financial instruments synergistic? -Comprehensive quantitative research based on content analysis
Source: PLoS One. 2024 Mar 7;19(3):e0298601. doi: 10.1371/journal.pone.0298601 (PMC10919601; doi:10.1371/journal.pone.0298601)
Supplement: S1 Data — (ZIP) [file pone.0298601.s001.zip › Data/地级市Z_F工作报告.zip!/▒▒╛⌐/└┤╫╘╣1⁄2╓┌║┼í╛┬φ┐╦╩2╛▌═°í┐_2015.docx]

——2015年1月23日在北京市第十四届人民代表大会第三次会议上

北京市市长 王安顺

各位代表：

　　现在，我代表北京市人民政府，向大会报告政府工作，请予审议，并请市政协各位委员提出意见。

　　一、2014年工作回顾

　　2014年是首都发展史上不平凡的一年。2月26日，习近平总书记视察北京并发表重要讲话，明确了北京作为全国政治中心、文化中心、国际交往中心、科技创新中心的战略定位，提出了建设国际一流的和谐宜居之都的目标，对做好北京发展和管理工作、推动京津冀协同发展作出了重要指示，为我们做好新时期首都工作指明了方向。首都的科学发展翻开了新篇章，开启了新航程。

　　一年来，在党中央、国务院和中共北京市委的坚强领导下，在市人大及其常委会的监督和支持下，我们努力提高思想认识，积极转变发展理念，扎实做好稳增长、促改革、调结构、惠民生各项工作，实现了经济社会持续稳步发展。全市地区生产总值比上年增长7.3%；一般公共预算收入增长10%；城乡居民人均收入分别实际增长7.2%和8.6%；城镇登记失业率1.31%；居民消费价格上涨1.6%；万元地区生产总值能耗、水耗、二氧化碳排放量预计分别下降5.5%、4%和6%，较好地完成了市十四届人大二次会议确定的目标任务。主要做了以下工作：

　　(一)着力激发市场活力，重点领域改革取得新突破。认真落实全面深化改革路线图和时间表，完成了110项年度重点改革任务。深化行政审批制度改革，取消和下放70项审批事项，公布市级投资项目审批事项清单，总结推广优化审批流程试点经验，实施“先照后证”、“三证合一”。出台政府向社会力量购买服务的实施意见。落实“营改增”扩围，总减税376.6亿元。制定实施全面深化市属国资国企改革的意见，启动国企分类监管、分类考核和董事会规范建设试点，国有企业重组改制步伐加快，北汽股份等3家企业上市，市属企业利润增长14.4%。推进公共设施建设市场化，市政基础设施试点引入的社会资本来源落实七成。成功发行地方政府债券105亿元。平稳实施阶梯水价、公共交通票制票价改革，调整非居民电价、气价、热价，提高排污收费标准，建立了水环境区域补偿制度。整体推进考试招生制度改革，义务教育免试就近入学取得突破。深化医药卫生体制改革，落实城乡居民大病保险制度，公立医院综合改革稳步推进。统筹抓好农村改革，集体经济组织产权制度改革、集体土地确权等取得新进展，“一绿”地区城市化建设试点全面启动。一系列放权、松绑、减负、惠民的改革“组合拳”，有效激发了发展活力和动力。

　　(二)深入推进结构调整和功能疏解，提质增效升级迈出坚实步伐。制定实施加快全国科技创新中心建设的意见，发布促进科技成果转化和协同创新的系列政策，中关村示范区先行先试深入推进，示范区企业总收入3.57万亿元、增长17.2%，全市技术合同成交额增长10%，发明专利授权量超过2万件、位居全国首位。积极促进产业结构优化升级，战略性新兴产业对规模以上工业增长的贡献率、高端服务业对地区生产总值增长的贡献率均达到六成左右。制定实施调结构转方式、发展高效节水农业的意见，都市型现代农业发展向好。成功举办第三届京交会，服务贸易进出口总额增长15.4%，实际利用外资90.4亿美元、增长6.1%。接待旅游人数2.6亿人次，旅游收入增长8%。顺利完成第三次经济普查。2019年北京世界园艺博览会获得国际最高级别的专业博览会举办资格，正式进入全面筹备阶段。世界知识产权组织中国办事处、亚洲基础设施投资银行落户北京。对口支援和帮扶协作年度任务顺利完成。

　　积极落实京津冀协同发展战略，下决心疏解非首都核心功能。制定实施新增产业的禁止和限制目录，关停退出一般制造业和污染企业392家，搭建了30个产业疏解合作平台，推进产业转移疏解项目53个，拆除中心城商品交易市场36个。与天津、河北分别签订了合作框架协议和备忘录，实施交通、生态环保、产业三个重点领域率先突破工作方案，北京新机场开工建设，首钢曹妃甸、张承生态功能区、中关村示范区与滨海新区合作等重点工作取得进展，中关村企业在天津、河北累计设立分支机构1532个。

　　(三)努力破解发展难题，城市服务管理水平稳步提升。制定实施人口规模调控工作方案，以功能、产业疏解带动人口疏解，全市常住人口增速进一步放缓，完成了人口调控年度任务。严格执行大气污染防治条例，全面落实清洁空气行动计划各项措施，西北热电中心建成投用，改造燃煤锅炉6595蒸吨，压减燃煤280万吨，淘汰老旧机动车47.6万辆，基本淘汰黄标车，完成平原百万亩造林主体工程。着力推进垃圾污水治理，全市生活垃圾资源化率提高了2.1个百分点，再生水年利用量增长了7.5%。南水北调中线一期工程建成通水，首都水资源严重短缺状况得到缓解，保障能力明显增强。持续开展交通治堵，4条轨道交通新线建成通车，完成100公里道路微循环畅通工程，交通运行基本平稳。启动石景山城市管理体制改革试点，网格化社会服务管理体系覆盖90%以上的社区村庄。大力开展环境综合整治，拆除违法建设1531万平方米，环境面貌和秩序进一步改善。城乡结合部50个重点村回迁安置房建设顺利推进，新型农村社区试点有序开展，588个村庄启动美丽乡村建设，农村人居环境不断改善。

　　(四)持续改善民生，首都和谐稳定的良好局面进一步巩固。为群众办的27件重要民生实事全面落实。完善就业创业政策体系，积极帮扶重点困难群体就业，城镇新增就业42.7万人。稳步提升社会保障待遇水平，健全社会救助政策体系，104个街乡镇养老照料中心建设顺利推进。积极改善群众住房条件，保障性住房竣工10.7万套、新开工10.1万套，实施棚户区改造项目205个，完成老旧小区综合整治1608万平方米、农宅抗震节能改造9.1万户。促进义务教育均衡发展，新增城乡一体化学校20所、学前教育学位1.6万个。成立30个区域医联体，市属医院全部实现分时段就诊。制定实施促进文化消费的意见，积极发展体育事业，群众文化体育生活更加丰富多彩。认真做好新时期民族、宗教、对台、侨务工作，积极推动妇女儿童、残疾人事业发展。强化食品药品监管，健全安全生产隐患排查治理和安全责任体系，深入开展矛盾纠纷排查化解，统筹反恐与维稳，扎实做好打防管控各项工作，维护了社会和谐稳定。进一步加强军政军民团结，驻京部队在首都改革发展稳定中发挥了重要作用。

　　(五)巩固和拓展党的群众路线教育实践活动成果，政府自身建设取得新进展。全面落实市政府党组教育实践活动整改方案，制定70项工作制度，174条具体问题整改全部落实，“四风”问题得到有效遏制。出台市政府信息公开规定，首次公开政府全口径预算，自觉接受社会监督。“三公”经费支出预算降低12.7%，市级节庆论坛展会活动的财政经费压缩40%。围绕重大政策措施落实，认真开展行政监察和跟踪审计。严格落实党风廉政建设责任制，开展腐败浪费行为专项整治，严肃查处各类违纪违法案件。

　　深入推进社会主义民主法治建设，全面执行市人大及其常委会的各项决议和决定，自觉接受人大工作监督、法律监督和政协民主监督，认真听取各民主党派、工商联、无党派人士和人民团体意见。办理市人大代表议案、建议1152件，政协建议案、提案1024件，提请市人大常委会审议通过地方性法规草案6项，制定、修改、废止政府规章22项。

　　各位代表，申办2022年冬奥会是党中央、国务院作出的重大决策。按照中央申办工作领导小组的统一部署，我们成立了冬奥会申办委员会，确定了“以运动员为中心、可持续发展、节俭办赛”的理念，完成了国际奥委会的调查问卷答复和视频答询，成功入围候选城市。积极参加国际奥协大会、国际奥委会第127次全会等重要活动，通过了7个国际冬季单项体育组织的场馆认证，向国际奥委会提交了申办报告。广泛开展冬季运动和冬奥文化宣传活动，为申办工作营造了良好氛围。

　　各位代表，在党中央、国务院的坚强领导下，在各有关方面的大力支持下，我们认真履行首都职责，圆满完成了新中国成立65周年庆祝活动和亚太经合组织第二十二次领导人非正式会议相关服务保障工作。两项重大活动服务保障任务的顺利完成，是我们在做好“四个服务”方面交出的一份合格答卷，同时也有力促进了全市各项工作，留下了宝贵的物质和精神财富，更加坚定了我们做好首都工作的信心和决心。

　　各位代表，过去一年的成绩来之不易，这是党中央、国务院和中共北京市委坚强领导的结果，是全市人民团结奋斗的结果。在这里，我代表北京市人民政府，向全市各族人民，向人大代表、政协委员，各民主党派、各人民团体和各界人士，向中央各部门、各兄弟省区市，驻京解放军和武警部队官兵，向关心支持北京建设发展的港澳台同胞、海外侨胞和国际友人，表示诚挚的感谢！

　　各位代表，在治理大气污染方面，去年全市上下付出了巨大努力，广大市民也积极参与、无私奉献。空气中细颗粒物年均浓度实际下降了4%，相比2014年政府工作报告提出下降5%左右的目标，还有一定差距。有关具体情况，市政府已向大会提交了大气污染防治条例实施情况的报告。

　　各位代表，冷静审视新阶段首都发展面临的新形势、新任务、新要求，我们清醒地看到，全市经济社会发展中还存在着不少矛盾和问题，政府工作还有不足和差距。主要是：人口规模调控难度大，资源环境约束趋紧，大气污染、交通拥堵治理需要付出长期艰苦的努力；城乡结合部建设亟待加强，遏制违法建设、非法经营等行为仍需加大力度，提高城市管理的法治化、精细化水平需要下更大的功夫；转方式、调结构、稳增长任务仍然十分艰巨，改革创新有待深化和突破；教育、医疗、文化等公共服务保障能力不足与资源配置不均衡并存，安全生产、食品安全等方面还存在隐患，保障改善民生任务繁重；在一些政府部门及工作人员当中，作风不实、效率不高、不作为、乱作为等现象不同程度存在，违反党纪政纪问题、腐败问题时有发生，改进作风和勤政廉政建设需要常抓不懈。问题所在就是努力的方向，人民期待就是工作的目标。我们将直面问题、敢于担当，履职尽责、扎实工作，决不辜负人民的期望。

　　二、2015年主要任务

　　今年是全面深化改革的关键之年，是全面推进依法治国的开局之年，也是全面完成“十二五”规划的收官之年，做好今年的工作意义重大。当前，我国经济发展步入新常态，新常态的主要特征和趋势性变化在首都经济中表现得比较突出和明显。推动首都科学发展，必须把适应经济发展新常态、落实“四个中心”的新定位、建设国际一流的和谐宜居之都的新目标，贯穿于全市各项工作之中，一步一个脚印，扎扎实实向前推进，不断取得新成效。

　　今年政府工作的总体要求是：深入贯彻落实党的十八大、十八届三中、四中全会和中央经济工作会议精神，深入学习贯彻习近平总书记系列重要讲话和对北京工作的重要指示精神，主动适应经济发展新常态，坚持稳中求进工作总基调，牢牢把握首都城市战略定位，积极落实京津冀协同发展战略，更加注重改革创新，更加注重经济发展的质量和效益，更加注重生态文明建设，更加注重城市精细化管理，更加注重保障和改善民生，更加注重法治政府建设，推动首都经济社会持续健康发展，向着国际一流的和谐宜居之都迈出坚实步伐。

　　全市经济社会发展主要预期目标是：地区生产总值增长7%左右；城镇登记失业率控制在3%以内；城乡居民人均收入实际增长7%；居民消费价格涨幅控制在3.5%左右；一般公共预算收入同口径增长7%以上；全社会研发支出占地区生产总值比重6%左右；万元地区生产总值能耗、水耗和二氧化碳排放量分别下降2%、4%和2.5%，空气中细颗粒物年均浓度下降5%左右。

　　重点抓好以下工作：

　　(一)积极推动京津冀协同发展。推动京津冀协同发展，是党中央、国务院在新的历史条件下作出的重大战略决策，也是我们适应新常态、落实新定位、迈向新目标的必由之路。要认真对接和落实京津冀协同发展规划纲要，牢固树立“三地一盘棋”的思想，主动担当、主动作为，推进协同发展实现良好开局。

　　加快疏解非首都核心功能。严格控制增量，有序疏解存量，对不符合首都城市战略定位的功能和产业，逐一列出清单，拿出具体方案，尽快组织实施，确保取得实质性进展。严格执行新增产业的禁止和限制目录，疏解一般制造业，禁止在首都功能核心区新建扩建制造业。加快疏解动物园地区批发市场、大红门地区批发市场、天意小商品批发市场，对其他区域性批发市场逐步制定调整疏解计划。积极推动部分教育、医疗等社会公共服务功能向外转移和疏解，促进京津冀区域教育合作和人员交流，探索共建大学新区、研发新区、创业园区和职教园区；深化医疗卫生领域合作，共建一批高水平的护理医院和康复医院；鼓励有实力的养老服务机构在周边地区建设养老服务基地，提高区域间公共服务均等化发展水平。

　　率先突破三个重点领域。制定实施交通一体化、生态环保、产业对接协作年度任务项目清单，尽快取得新的成效。抓好京沈客专、京张铁路、京台高速等跨省市铁路、高速公路和国省干线公路建设，打通一批断头路，促进区域重点城市公交“一卡通”。以张承生态功能区建设为重点，深入实施水源保护林、风沙源治理等区域生态保护与发展合作项目，推进京津保中心区过渡带生态建设。立足合作共赢，推动曹妃甸协同发展示范区、天津滨海-中关村科技园区建设。加快新机场征地拆迁和外围设施建设，推动临空经济合作区规划编制和实施工作。

　　探索建立协同发展机制。做好跨行政区、跨领域规划对接，推动建立统一的生态环境规划、标准、监测、执法体系，积极探索共建产业园区税收、节能减排等方面的利益分配和责任分担机制。逐步建立三地统一的企业产权、知识产权、林权矿权等市场化流转制度，推动制定跨区域的技术、人才等要素流动和优化配置的政策措施，促进金融、旅游等资源共享，加快区域市场一体化进程。

　　推动形成协同创新共同体。强化协同创新支撑，以中关村和共建园区为依托，推动建设跨省市科技创新平台，支持开展联合攻关，打造产学研结合的科技创新链条，构建分工合理的创新发展格局。完善区域创新体系，联合组建产业技术创新战略联盟，完善信息共享、标准统一的技术交易服务体系，推进一体化的技术市场建设。加强科技人才和科技管理人才的联合培养，搭建人才信息共享平台，健全跨区域人才流动机制。

　　(二)全面深化改革开放。深化改革开放，是适应新常态、落实新定位、迈向新目标的动力源泉。要处理好政府与市场的关系，协调推进各领域改革，让发展活力竞相迸发。

　　继续简政放权。政府要勇于自我革命，把该放的放到位，把该给的给到位，把该管的管到位，给市场主体创业创新留出空间、搭好舞台。深入推进行政审批制度改革，再精简200项以上审批事项，不再保留非许可类审批事项，依法加强事中事后监管。深化投资项目审批流程改革，市级具有审批职能的部门进驻政务服务中心开展工作，实现审批规范化和集中化。深化商事制度改革，积极推进“先照后证”，落实多部门联办机制，为企业准入提供更多便利。全面推广使用组织机构代码，推动部门间数据互联互通，加强社会信用体系建设。

　　稳步实施财税金融改革。贯彻落实预算法，深化预算管理制度改革，扩大滚动项目预算编制范围，实行全过程预算绩效管理。做好“营改增”扩围，清理规范税收优惠政策。适时调整非居民气价、热价，研究推行居民阶梯气价政策，积极推进医疗服务价格改革。落实加快建设国家科技金融创新中心意见，推动设立中关村民营银行，支持私募股权基金、创业投资基金、天使投资发展，深化科技信贷、科技保险、外汇管理等创新试点，支持全国中小企业股份转让系统、北京股权交易中心建设发展。完善地方金融风险防控体系，有效化解金融风险。

　　增强各类市场主体活力。出台市属国资国企改革实施规划和配套政策，妥善解决国有企业历史遗留问题，推动市属企业与中央企业、中关村高新技术企业合作发展，完善法人治理结构，提高国有资本证券化率。健全国资监管体制，以管资本为主加强国资监管，完善国有企业负责人长效激励和约束机制。调整优化国有经济布局，把国有资本更多集中到公共服务、基础设施建设、生态环境保护以及战略性新兴产业等领域。扩大公共领域市场化试点，通过政府和社会资本合作等多种方式，鼓励社会资本进入轨道交通、镇域供热、水环境治理等领域。发挥首都金融优势，支持科技创新型中小企业发展。实施普遍性降费，减轻企业特别是小微企业负担，为非公有制经济发展创造良好环境。

　　全方位扩大开放。立足国际交往中心定位，服务国家对外交往大局，主动融入国家“一带一路”战略，深化对外交流与合作。贯彻落实支持外贸稳定增长、促进贸易便利化政策措施，加快外贸转型升级示范基地和出口产品质量安全示范区建设，推进天竺综保区、平谷国际陆港建设发展，支持“双自主”企业扩大出口，促进跨境电子商务发展，培育新的外贸增长点。扩大服务业开放，大力发展服务外包，推动服务贸易健康发展。研究制定促进境外投资发展的实施方案，支持企业走出去。深化京港、京澳、京台合作，发挥侨务对外交流渠道优势。组织实施好年度援建项目，深入推进对口支援新疆、西藏、青海等地区工作，扎实做好对口帮扶协作。

　　(三)深入实施创新驱动发展战略。创新驱动是适应新常态、落实新定位、迈向新目标的根本举措。要立足全国文化中心、科技创新中心的定位，持续推进体制机制改革，促进科技文化创新不断取得新突破。

　　加快全国科技创新中心建设。深化科技体制改革，完善创新体系，聚集创新人才，提升自主创新能力，在创新型国家建设中发挥示范引领和辐射带动作用。推进科技成果使用权、处置权和收益权改革，加快制定符合创新规律的财政科研项目和资金管理办法，让人才、资金、技术、管理等创新要素充分活跃起来。强化企业技术创新主体地位，实施研发机构建设、研发投入引导、创新环境营造三项工程，支持企业自建或与高校院所共建研发机构、中试基地和成果转化基地。积极搭建创业创新平台，发挥好首都科技条件平台、科技大数据平台、技术创新联盟等作用，推动产学研用协同创新。全面对接国家重大科技专项和重大科技基础设施建设，服务国家创新战略。落实技术创新行动计划、“京校十条”和“京科九条”，争取一些关键领域和共性技术取得突破，打通技术转移、产品中试和推广应用三个环节，建设面向全国的新技术、新产品采购平台，推动科技成果转化和产业化。更好地发挥中关村主战场作用，深入落实“1+6”和“新四条”，积极争取国家新一轮试点政策，完善一区十六园统筹发展机制，推动示范区高水平发展。

　　提升首都文化软实力。大力弘扬社会主义核心价值观，广泛开展中国梦、依法治国等主题教育，深入推进“讲文明、树新风”活动和群众性精神文明创建活动，进一步提升市民文明素质和城市文明程度。做好纪念中国人民抗日战争暨世界反法西斯战争胜利70周年有关活动的服务保障工作，弘扬爱国主义精神。充分发挥道德模范、“北京榜样”的示范作用，扎实做好思想道德建设。构建现代公共文化服务体系，出台加强基层公共文化建设意见，加快建设北京市文化活动中心等文化基础设施，引导文学艺术繁荣发展，推出一批精品力作。加强优秀传统文化保护、传承与发展，做好“三山五园”、周口店遗址、长城文化带的保护利用，加强历史文保区修缮整治与改造更新，保护好历史文化名城这张“金名片”。全面推进文化体制机制创新，深化国有文化单位分类改革，建立已改制院团的长效扶持机制，完善文化投融资体系，支持北京文化产权交易中心建设发展，培育多层次文化产品和要素市场。落实文化创意产业功能区建设发展和产业提升规划，抓好国家文化产业创新实验区、国家数字出版基地、中国乐谷、怀柔影视产业示范区建设，推动文化创意和设计服务与科技、旅游、体育等相关产业融合发展。扩大对外文化交流，推动文化贸易发展和文化走出去，增强首都文化的国际影响力。扩大体育产品和服务供给，加快发展体育产业。全力办好2015年世界田径锦标赛。

　　厚植创业创新文化。统筹做好人才引进、培养和使用，推进人才特区建设，落实“千人计划”和“海聚工程”，实施“四个一批”文化人才培养工程，完善创新人才评价和激励机制，让创新人才愿意来、留得住。鼓励创新型和专业性孵化器发展，加大中介机构和社会组织培育力度，加快构建覆盖创新全链条的服务体系。出台促进知识产权转化运用的政策措施，建设全国知识产权运营公共服务平台，依法惩处侵权行为。落实国家质量发展纲要，加强品牌建设，深化标准创新试点，建设国家技术标准创新基地。实施中关村“创业中国引领工程”和公众参与创新行动计划，营造有利于科技、文化创新的社会氛围。

　　(四)着力构建“高精尖”经济结构。构建“高精尖”经济结构是适应新常态、落实新定位、迈向新目标的核心任务。要做好经济结构调整的加减乘除法，优化三次产业内部结构，大力发展总部经济，加快形成创新引领、技术密集、价值高端的经济结构。

　　积极培育高端产业发展新优势。做大做强战略性新兴产业，加快新一代信息技术、生物、新材料、航空航天、高端装备制造业创新发展。支持云计算与物联网、移动互联网等融合发展，鼓励用信息技术重构和整合传统产业链条，加速催生新技术、新产品、新模式、新业态。制定实施促进生产性服务业发展的意见，抓好石景山国家服务业综合改革试点和中关村现代服务业试点，发展互联网金融、大数据金融，提升信息服务业、科技服务业发展水平。加快推进北京保险产业园建设，促进现代保险服务业发展。制定实施提高生活性服务业品质三年行动计划，推行连锁经营、集中配送等现代流通方式，引导生活性服务业品牌化、特色化、规模化发展。大力发展节能环保朝阳产业，推广应用新能源技术和节能环保产品，推动合同能源管理、污染第三方治理，支持太阳能、风能、生物质能等新能源开发利用。加快配套设施建设，新能源汽车配置指标增加到3万辆，推动新能源汽车产业发展。

　　巩固提升以消费为主导的内需结构。大力实施养老健康家政、信息、旅游、住房、绿色、教育文化体育等6大领域消费工程，积极促进大众消费。培育北斗导航产品、云终端、智能家电等消费热点，进一步扩大信息消费规模。支持网络零售发展，扩大电子发票试点范围，培育线上线下相融合的消费新模式。办好惠民文化消费季等系列活动，促进文化和体育消费。落实国民旅游休闲纲要，执行职工带薪休假制度，打造以特色小镇、古村落为重点的休闲度假品牌，提升旅游休闲消费。做好投资促进各项工作，以政府投资引导带动社会投资，重点投向生态环境、基础设施、民生改善等领域，抓好重大项目建设，提高投资效率。

　　打造“六高四新”高端产业主阵地。细化高端产业功能区定位，健全重大产业项目统筹协调机制，走内涵集约发展道路。着力完善“六高”功能区产业、金融、生活配套等支撑体系，合理控制开发强度，促进北京经济技术开发区转型升级、创新发展，提升金融街、商务中心区发展水平。加快丽泽金融商务区等“四新”功能区建设发展，抓好雁栖湖生态发展示范区后续建设和利用，尽快积聚实力、形成规模。

　　(五)优化完善城市发展格局。完善城市发展格局是适应新常态、落实新定位、迈向新目标的重要支撑。要坚持城乡发展一体化，深化区县功能定位，划定城市增长边界和生态红线，形成生产、生活、生态空间的合理结构。

　　充分发挥规划龙头作用。创新规划理念，加强顶层设计和统筹力度，切实提高规划的前瞻性、系统性和科学性。高质量完成城市总体规划修改，以人口资源环境承载能力为底线，统筹功能疏解、人口控制、用地减量、空间优化等目标任务，促进城市可持续发展。拓宽公众参与渠道，高水平做好“十三五”规划和各专项规划编制工作，认真对接国家“十三五”规划，深入研究重点难点问题，推动经济社会发展、城乡、土地、人口、环境等“多规合一”。强化规划的严肃性、权威性，从严实施，红线管理，确保一张蓝图绘到底。

　　提升城市建设特别是基础设施建设质量。切实树立国际一流标准，在功能区块、市政公用设施、单体建筑建设等方面科学设计、精心施工，逐步形成适度超前、相互衔接、满足未来需要的功能体系。抓好水电气热、通信等市政设施规划建设，开工建设陕京四线工程，加强地下管线综合管理，做好油气输送管道隐患排查整治，增强城市运行保障能力。完善相关制度措施，科学适度地开发利用好城市地下空间。建成密云水库调蓄等工程，完成南水北调后续配套规划，建设改造300公里供水管网，做好水质监测和运行管理，实施农村饮水提质增效工程，让城乡居民吃上放心水。全力推进节水型城市建设，推广使用节水型器具，引导全社会节约用水。全面完成垃圾、污水处理设施建设三年任务，生活垃圾资源化率由52%提高到55%，再生水年利用量由8.6亿立方米增加到9.5亿立方米。下更大力气加快建设污泥处理处置设施，已确定的项目明年上半年全部建成投运，基本实现全市污泥无害化处置，以“零容忍”的态度严肃查处污水、污泥违法排放等行为。加强水域生态保护，持续治理中小河道。继续实施下凹式立交桥泵站改造和雨洪利用工程，加快气象现代化基础设施建设，完善城市综合防灾减灾体系。

　　多层级推进新型城镇化。坚持城镇发展与产业支撑、中心城人口疏解相统一，推动建设重心向郊区转移，形成多点支撑、均衡协调的城市发展格局。编制实施城乡结合部建设三年行动计划，基本完成50个重点村建设任务，扩大“一绿”地区城市化建设试点，抓好“二绿”地区统筹城乡建设用地试点工作，推动城乡结合部减人增绿。加快城市副中心建设，完成通州核心启动区基础设施建设任务，推进北京环球主题公园等重点项目，抓好国家级新型城镇化试点工作。推进顺义、亦庄-大兴、昌平、房山等综合性新城建设，提高教育、医疗等公共服务水平，承接中心城人口、功能疏解转移。吸引社会资本参与小城镇建设，打造一批功能定位清晰、配套设施完善、生态环境优良的特色小城镇。

　　深入推进新农村建设。以“土地流转起来、资产经营起来、农民组织起来”引领农村改革与发展，引导城市现代生产要素向农业农村流动。健全土地资源节约集约利用制度体系，严守耕地保护红线。加快农村土地承包经营权确权登记颁证，支持发展农业适度规模经营。积极争取集体经营性建设用地入市试点，促进集约高效利用。深化集体林权制度改革。推动集体经济组织产权制度改革向乡镇级拓展，健全农村产权流转交易市场，加强集体资产监管。培育创建一批市级示范合作社。推进农业调结构转方式，划定80万亩粮田和70万亩菜田，抓好“米袋子”、“菜篮子”重点工程，加快发展高效节水农业，提升农田的生态涵养功能。继续推动美丽乡村建设，抓好48个新型农村社区试点，做好重点传统村落修缮保护，搬迁山区农民9050人。精准帮扶低收入村和低收入户，着力促进农民增收。

　　大力支持薄弱地区发展。继续实施第二阶段城南行动计划，加快南部地区公共服务、基础设施、生态环境等重点项目建设。落实首钢老工业区改造调整和建设发展的政策措施，引领带动西部地区转型发展。积极推动生态涵养发展区绿色发展，加快提升乡村旅游业发展水平，发展生态服务型经济。推进延庆、密云国家主体功能区建设试点示范，支持门头沟创建国家可持续发展试验区。制定2019年北京世界园艺博览会行动纲要，完成园区综合规划及周边基础设施规划方案，做好园区拆迁和土地腾退。

　　(六)全力破解城市发展难题。破解发展难题是适应新常态、落实新定位、迈向新目标的迫切需要。我们必须以坚韧不拔的毅力、坚定不移的决心，加大力度，综合施策，坚决打好治理“城市病”的攻坚战。

　　严格控制人口规模。全面实施人口规模调控方案，落实区县调控责任，强化依法管理，实现常住人口增速明显下降。出台居住证制度，研究制定积分落户政策。推动制定房屋租赁管理条例，开展新一轮地下空间三年整治，依法治理群租房，加强人口数据信息动态监测，做好流动人口的服务与管理。合理运用财政、税收、价格等经济手段，就地淘汰低端、低效、高能耗产业，关停300家制造、锻造、小家具等领域的污染企业，逐步清退镇村工业大院，以产业疏解带动人口疏解。

　　持续抓好大气污染治理。严格执行环境保护法和大气污染防治条例，全面实施压减燃煤、控车减油、治污减排、清洁降尘措施。加大财政资金支持力度，核心区全面实现无煤化，城六区基本取消燃煤锅炉，农村地区新增减煤换煤120万吨。淘汰老旧机动车20万辆，水泥产能由700万吨压缩到550万吨，推进绿色建筑发展和住宅产业化，开征施工工地扬尘、挥发性有机物的排污费。推动编制实施区域大气污染防治中长期规划，完善预报预警、科技治污、区域联防联控机制，加大空气重污染应急措施的实施力度。落实企业环境责任，动员全社会共同参与雾霾治理。全面完成平原百万亩造林任务，统筹做好新增林木、原有林地的流转和管护，新增城市绿地600公顷，建设市级健康绿道200公里。开展农用地、废弃地和山区生态修复，完成生态清洁小流域治理400平方公里。抓好农业源污染减排，促进秸秆、粪污等农业废弃物资源的循环利用。

　　着力缓解交通拥堵。坚持优先发展公共交通，制定实施新一轮综合缓堵措施，加快建设安全、便捷、高效、绿色、经济的综合交通体系。加大轨道交通建设力度，开工建设4条新线，开通14号线中段、昌平线二期。完善中心城道路网络，推进广渠路二期、苹果园交通枢纽等项目。建设12个停车管理示范小区、30条示范街，推进驻车换乘停车场建设。加大停车治理力度，严格停车执法，规范停车收费。围绕交通拥堵重点区域，加强交通秩序整治。实施公交站台改造、疏堵项目、规范自行车道“三个一百”工程，施划公交专用道30公里，增开微循环公交，合理规划人行步道，公共租赁自行车达到5万辆，方便市民出行。

　　不断加强城市精细化管理。深化城市管理体制改革，总结推广石景山试点经验，出台街道社区管理体制改革意见，推进城市管理重心下移、力量下沉。制定一批城市治理和建设发展方面的地方标准，新建500个智慧社区，加快推进社会服务、城市管理、社会治安三网融合，提高管理效率。保持“拆违打非”高压态势，拆除违法建设1500万平方米，确保新增违法建设零增长。集中力量整治城乡结合部环境，提升重点地区、重要大街环境质量，抓好痼疾顽症的专项整治与日常治理，让城市更加整洁有序。

　　(七)切实保障和改善民生。保障和改善民生，是政府工作的出发点和落脚点。要把更多资金用到民生领域，着力保障基本、创新机制、强化治理，努力提高人民群众生活水平。

　　做好就业和社会保障工作。以创业带动就业为重点，强化政策集成，落实大学生就业促进计划和创业引领计划，做好教育、培训、资金支持等服务，帮助更多青年实现创业梦想。研究制定城乡自谋职业、灵活就业社保补贴政策，加强专项帮扶，促进结构性失业人员、就业困难人员和农村转移劳动力就业。做好社会保障制度整合衔接，制定机关事业单位与企业职工养老保险并轨的具体办法，推进医疗保险付费方式改革，完善社保待遇标准联动调整机制。深入开展“救急难”工作，统一城乡低保标准，扩大社会福利覆盖面。积极应对老龄化社会带来的挑战，落实老年人权益保障法，出台居家养老服务条例相关配套措施，推动公办养老机构改革，支持社会力量兴办服务机构和企业，加快养老服务业和健康服务业发展，新建40个街乡镇养老照料中心。坚持男女平等基本国策，保障妇女儿童权益。加强无障碍设施建设，做好残疾人基本公共服务。支持慈善事业发展。

　　推进教育改革发展。制定实施教育综合改革意见，进一步巩固义务教育免试就近入学改革成果，坚持学区制、九年一贯制和教育集团等做法，实现三年新建、改扩建200所中小学的目标。加强教师队伍建设，落实义务教育学校校长和教师交流轮岗制度。实施第二期学前教育三年行动计划，新建、改扩建一批幼儿园。抓好城乡一体化高中建设，推动普通高中多样化发展。深化课程改革，支持中小学开展课外体育、艺术、科技活动，推进素质教育。加强残疾人融合教育。开展职业教育与普通高校联合办学试点，在高校实施高水平人才培养、高质量就业创业、“高精尖”创新中心建设三项计划，提升人才培养水平和创新服务能力。

　　提高人民群众健康水平。深化公立医院改革，全面推进医药分开，落实医师多点执业政策，鼓励和引导社会力量举办医疗机构。完善基层医疗卫生服务体系，增强基层医疗机构的康复功能，加强全科医生培养，健全分级诊疗制度，引导患者有序就医。加快区域医联体建设，基本建成郊区县10个区域医疗中心，推进北京国际医疗服务区建设，开展中医健康乡村建设试点，推动优质医疗资源均衡配置。健全传染病防控和救治机制，加强慢性病和重性精神疾病的管理，提升公共卫生安全水平。实施控制吸烟条例。做好计划生育服务管理工作。制定实施新一轮全民健身计划，不断提高市民身体素质。

　　改善群众住房条件。加大住房保障力度，通过新建、收购、租赁和货币补贴等多种方式，筹集各类保障房10.5万套，确保完成“十二五”期间新增100万套的任务。加强统筹联动，抓好资金保障、政策制定、对接安置等关键环节，分类推进棚户区改造，累计完成8万户搬迁任务。加快中心城棚户区和文保区改造，启动实施天坛周边简易楼、前门东区、菜园街和光源里等重点项目，务必取得突破性进展。实施1150万平方米老旧小区综合改造，完成8万户农宅抗震节能改造任务。

　　加强和创新社会治理。出台深化首都社会治理的若干意见，深化基层组织和部门、行业依法治理，支持各类社会主体自我约束、自我管理，发挥市民公约、乡规民约、行业规章和团体章程等社会规范作用。完成第九届社区居委会换届选举，全面推进社区民主协商。加强社区工作人员队伍建设，开展老旧小区自我服务管理和村级社会服务管理试点，完成200个“一刻钟社区服务圈”、100个社区规范化示范点建设任务。健全“枢纽型”社会组织工作体系，支持社会组织依法参与社会事务，推进行业协会、商会与行政机关脱钩，扩大政府购买社会组织服务规模。深化社会动员试点，推进志愿服务常态化、规范化。整合优化非紧急救助综合服务平台功能，健全社会监督评价体系，引导市民有序参与社会治理。

　　全力维护社会和谐稳定。实施安全发展战略，落实安全生产“一岗双责、党政同责”制度，做好消防、交通、生产经营等领域的隐患排查治理，抓好安全生产责任保险制度试点，加强人员密集场所和大型活动的安全管理，坚决防范遏制重特大事故。完善食品药品安全体系，强化全链条风险监测控制。加强突发事件信息预警和应急准备，提高机动处置能力。抓好社会矛盾源头治理，完善人民调解、行政调解、司法调解联动工作体系，健全重大决策社会稳定风险评估机制，畅通群众诉求表达渠道。全面实施“阳光信访”，提高初信初访化解率。健全网络安全监管机制，创新立体化社会治安防控体系，抓好治安重点地区和突出治安问题的常态化治理，提升反恐防恐能力，深化“平安北京”建设。贯彻落实中央民族工作会议精神，深入推进首都民族团结进步事业发展，发挥宗教界人士和信教群众在促进经济社会发展中的积极作用。支持国防和军队建设，完善军民科技深度融合机制，做好双拥共建和转业安置工作，形成维护首都和谐稳定的强大合力。

　　各位代表，2022年冬奥会申办工作正处在关键阶段。我们要严格按照国际奥委会的要求，精心做好国际奥委会评估团考察接待工作，高质量完成主题陈述、场馆考察等任务。积极参加重要国际体育会议、冬季国际体育赛事，密切与国际奥委会及国际体育单项组织联系，高水平做好向国际奥委会全会的最终陈述。广泛开展冬季体育运动，大力推进冰雪体育进校园、进公园、进商业园，深化冬奥文化宣传，营造良好的申奥氛围。要充分借鉴2008年奥运会的成功经验，调动一切积极因素，全力以赴做好申办工作。

　　三、加快建设法治政府

　　建设法治政府是全面落实依法治国基本方略、推进国家治理体系和治理能力现代化的重要内容，也是建设好、发展好、管理好北京这座超大型城市的重要保障。我们要认真贯彻全面推进依法治国若干重大问题的决定和中共北京市委贯彻落实意见，加快建立权责统一、权威高效的依法行政体制，建设职能科学、权责法定、执法严明、公开公正、廉洁高效、守法诚信的法治政府，为建设法治中国首善之区奠定坚实基础。

　　依法全面履行政府职能。完善各层级、各部门职能配置，理顺职责关系，规范政府职权目录，推动政府事权规范化，机构、职能、权限、程序、责任法定化、公开化。厘清政府与市场、社会的边界，全面推行政府权力清单制度，执行市级审批事项清单、投资项目审批事项清单和行政处罚权力清单。健全法治政府指标体系和评价体系，把区县政府依法行政工作纳入绩效考核。创新政府服务管理，更多运用市场化、社会化、信息化方式，努力提高办事效率和服务水平。

　　健全行政决策机制。推进重大行政决策程序地方立法，明确事项范围、程序和责任，把公众参与、专家论证、风险评估、合法性审查、集体讨论决定作为重大行政决策法定程序。规范重大行政决策出台前向人大报告的程序。推行政府法律顾问制度，建立法律顾问队伍。完善行政决策档案管理制度，建立重大行政决策绩效评估机制，加强对重大行政决策实施情况跟踪反馈和监督检查。完善决策过错认定标准和责任追究启动机制，落实重大行政决策终身责任追究制度及责任倒查机制。

　　做好政府立法和普法工作。加强对政府立法工作的统筹，完善法规起草和规章制定程序。制定行政调解工作办法、行政执法与刑事司法衔接规定，积极推进急救医疗服务、旅游等领域立法，加强民生改善、城市安全运行、生态环境保护等方面制度建设。完善立法后评估机制，建立常态化的法规规章清理机制。深入开展法治宣传教育，落实普法责任制。政府工作人员要带头学法守法、依法办事，引导全体市民自觉守法、遇事找法、解决问题靠法。健全公共法律服务体系，完善法律援助制度，保证人民群众获得及时有效的法律帮助。

　　严格规范公正文明执法。深化行政执法体制改革，构建横向有分工、纵向有层级，权责明确、边界清晰、衔接有序、传导有效的行政执法体系。深入推进综合执法，理顺城管执法体制，强化综合执法与行政管理、专业执法的协调与惩戒联动。加强行政执法队伍建设，严格实行行政执法人员持证上岗和资格管理制度，健全行政执法裁量权基准制度，加大对环境保护、食品药品、劳动保障、停车秩序、房屋租赁等关系群众切身利益的重点领域执法力度。针对不作为、乱作为、选择性执法等突出问题，切实加强行政监察和究责问责。加强执法监督，建立执法全过程记录制度，启动执法信息服务平台建设，推行行政执法公示制度，强化对投诉举报案件的督查督办和监督考核。

　　加强对行政权力的制约和监督。自觉接受人大工作监督、法律监督和政协民主监督。强化政府内部流程控制，健全层级监督工作机制，推进行政问责制度化、规范化。发挥行政复议监督纠错功能，认真做好行政应诉工作。加强审计工作，保障依法独立行使审计监督权，对公共资金、国有资产、国有资源和领导干部履行经济责任实行审计全覆盖。加大政府信息公开力度，推进预决算、公共资源配置、重大建设项目批准和实施、社会公益事业建设等领域的信息公开，接受人民群众和新闻舆论监督。

　　持之以恒转变作风。深入贯彻落实十八届中央纪委五次全会精神，坚定不移推进党风廉政建设和反腐败斗争。不断强化党性修养和规矩意识，严守党的政治纪律和政治规矩，坚决在思想上政治上行动上与党中央保持高度一致，坚决维护党中央权威，维护党的团结。深化“四风”整治，巩固和拓展党的群众路线教育实践活动成果，全面落实各项整改任务，切实兑现承诺。认真贯彻中央八项规定精神和国务院“约法三章”，控制行政成本，完成党政机关公务用车制度改革，确保“三公”经费只减不增。认真落实党风廉政建设主体责任，抓好职责范围内的党风廉政建设。各级领导干部要充分发挥表率作用，认真落实“三严三实”的要求，切实做到为民务实清廉，以上率下改进作风。要加强公务员队伍建设，优化队伍结构，健全考核制度，抓好培训，从严管理，全面提高公务员政治素质和业务能力。要大兴调查研究之风，全面推行机关联系基层、干部联系群众“双联系”制度，以良好的作风、优异的业绩赢得群众的支持和信任。

　　各位代表！新常态带来新机遇，新定位开启新征程。让我们更加紧密地团结在以习近平同志为总书记的党中央周围，在中共北京市委坚强领导下，团结依靠全市人民，开拓创新，真抓实干，全面完成今年经济社会发展和“十二五”规划各项目标任务，为加快建设国际一流的和谐宜居之都努力奋斗，为实现中华民族伟大复兴的中国梦作出新贡献！
